# Comparison of Survival and Development of Gypsy Moth *Lymantria dispar* L. (Lepidoptera: Erebidae) Populations from Different Geographic Areas on North American Conifers

**DOI:** 10.3390/insects11040260

**Published:** 2020-04-24

**Authors:** Melody A. Keena, Jessica Y. Richards

**Affiliations:** Northern Research Station, Forest Service, United States Department of Agriculture–51 Mill Pond Road, Hamden, CT 06514, USA; Jessica.Y.Richards@usda.gov

**Keywords:** host utilization, *Lymantria dispar*, gypsy moth, survival, development, conifers

## Abstract

Host utilization information is critical to managers for estimating the hosts at risk and potential geographic range for gypsy moths from different geographic origins. In this study, the development and survival of gypsy moths from all three subspecies on 13 North American conifers and three broadleaf hosts were compared. There was variation in the ability of gypsy moth larvae from different geographic origins to utilize (survive and develop on) key North American conifers. However, that variation was not consistent within gypsy moth subspecies, but instead was more consistent with populations from different origins being preadapted to utilize different hosts and having different biologic traits. Some Asian populations developed and survived well on some conifers while populations from Europe and North America gained weight faster and/or survived better than some Asian populations. Although development was slower and survival poorer on several of the conifers, first instar larvae were able to utilize conifers unless the needles were tough or feeding deterrents were present. Host phenology was also critical since the early instars fed preferentially on new foliage or buds. Gypsy moth larvae can utilize many hosts, so this makes it a very adaptable invasive species that warrants taking measures to prevent its spread.

## 1. Introduction

Generalist herbivores tend to be more widespread than specialists and the breadth of their diet can vary over their geographic range. However, some populations may become more specialized than others, especially if they are small or geographically isolated. Coevolutionary theory proposes that differences between herbivore populations have arisen through taxon-specific histories of reciprocal adaptations that the herbivore has with its native or local host plants. However, recent findings suggest that this may not be the case since expression of host defenses may not be correlated with phylogeny and herbivores are selecting hosts that they are preadapted to utilize [1]. Plants have multiple potential defenses—structural, phenological, chemical, and through associations with other organisms that protect them—and they can evolve independently, so herbivores must either adapt to any new defense or evolve phenotypic plasticity to deal with a range of defenses. The more variable the environment the herbivore finds itself in the more plastic phenotypes will be favored [2].

In order to deal with a range of host secondary compounds, generalist herbivores like the gypsy moth (*Lymantria dispar* L., Lepidoptera: Erebidae) have evolved the use of general biochemical mechanisms which would be advantageous when they invade new areas [3]. This is particularly important because host species and even genera vary geographically; there are no native tree species shared between North America and Eurasia in the temperate zone, although there are congeneric species [4]. The host in an invaded area will also be adapted to its specific herbivore community and may not have defenses which are effective against newly introduced herbivores, allowing them to utilize a potentially broader host range. Host choice is critical, in either the native or introduced range, because it can influence the herbivore population dynamics through effects on fecundity, developmental time, survival, and natural enemy avoidance.

There are three subspecies of gypsy moth that differ biologically and ecologically, both within and between subspecies, across the native range of this species. *Lymantria dispar dispar* L. is the European subspecies that was introduced to North America in 1896; the females are flightless except for those in northern and eastern parts of Europe [5]. *Lymantria dispar japonica* Motschulsky and *Lymantria dispar asiatica* Vnukovskij are collectively known as Asian gypsy moth, and most females are capable of strong directed flight and are attracted to lights at night [5,6]. *L. dispar asiatica* is found east of the Ural Mountains in Russia and throughout most of China and Korea, while *L. dispar japonica* is found in patches on the islands of Japan [7]. Since the 1990s, there have been several introductions of gypsy moths from Asia into North America [8]. Most of these introductions occurred when females from Asian populations flew to lights at night in Asian ports where they laid their egg masses on ships or their cargo [6]. These egg masses were subsequently transported to North American ports where the larvae hatched, dispersed, and found suitable foliage to establish. Federal managers in both the United States and Canada immediately put into place eradication protocols in the areas where they established, and in each case the eradication was deemed successful. This drastic response was considered warranted for several reasons: females of Asian populations have a greater dispersal ability than those of their European counterpart [9]; some Asian populations have lower chill requirement for eggs to hatch [10]; and, according to the literature, larvae from Asian populations feed on a broader range of hosts (including some conifers) than the established population [11,12]. Although there are ship inspection programs in place at most Asian ports, egg masses are still found on vessels entering North American ports.

North American *L. dispar dispar* larvae are highly polyphagous, with 148 host trees identified as highly susceptible hosts (category 1) out of a total of 449 tree species that larvae can feed and complete development on (categories 1 and 2, [13]). The two genera with the most susceptible species are *Quercus* and *Salix*, with the remainder of the highly susceptible species coming from 28 other tree genera (including *Larix*, a deciduous conifer). A review of gypsy moth host trees in Europe identified 300 species and *Quercus* was again the most preferred genus [14]. In both North America and Europe, the range of hosts the larvae utilize expands once they reach the fourth instar and can include several conifers, such as some pine, spruce, and hemlock [4]. One laboratory study using egg masses collected in Oregon, USA found that first instar larvae could complete development on *Abies concolor*, two North American *Larix* species, *Picea pungens*, *Pinus ponderosa*, and *Pseudotsuga menziesii,* but the gypsy moths used were not tested to see if they might be of Asian origin [15]. More recently it was reported that *L. dispar dispar* in Spain had completed development on and defoliated *Pinus radiata* plantations [16].

Asian gypsy moth (*L. dispar japonica* and *L. dispar asiatica*) larvae utilize many of the same host tree genera as *L. dispar dispar* and have been reported to feed on 316 species from 61 orders and 130 genera [17,18,19,20,21]. Asian gypsy moth is known to outbreak on different hosts throughout its range, including *Quercus-*, *Betula-*, or *Larix*-dominated forests and mixed broadleaf forests [19]. Four laboratory studies evaluating host utilization by Asian (and European in two cases) gypsy moths have been carried out: with larvae from Siberian Russia, Michigan, USA, and Germany on 20 European tree species (first 10 days of development, [22]); with larvae from France, Siberian Russia, and Beijing, China on 41 *Eucalyptus* species (development to pupation, [23]); with larvae from Hokkaido, Japan on 71 tree species (first 14 days of development, [24]); and with larvae from two Chinese populations on 16 hosts (development to pupation, [25]). These studies generally showed that the Asian populations evaluated could utilize many of these hosts, and some populations developed faster and survived better on hosts considered marginal for North American gypsy moths. One earlier study of larvae from several Asian and European source populations on *Pseudotsuga menziesii* (in the first 14 days of development) showed that there was considerable variation in the survival and development of larvae on this host and that the ability to utilize this host was not gypsy moth subspecies specific [26]. However, there has been no study published comparing the host utilization of Asian and European gypsy moths from several different countries on key North American conifers.

Host utilization information is critical to managers for estimating the hosts at risk and potential geographic range for Asian gypsy moths from different geographic origins in North America. Since the lists of hosts that Asian gypsy moth is known to feed on in other countries is long and no broad evaluation of North American hosts has been performed, it is difficult to evaluate how the hosts at risk in North America to the Asian and established gypsy moths may differ. This study compared the host utilization of gypsy moths from all three subspecies, originating from multiple countries, on 13 key North American conifers and three broadleaf hosts. Variation between and/or within a subspecies in host utilization was assessed using survival and developmental data (either to 14 days or to adult with reproductive traits also evaluated). A discussion of regulatory implications and possible reasons for observed differences is included.

## 2. Materials and Methods 

### 2.1. Gypsy Moth Populations

Information on subspecies and approximate location (latitude and longitude) of source populations, for the six *Lymantria dispar* geographical populations used in these studies, is given in Table 1. The populations from the United States and Greece) served as the *Lymantria dispar dispar* controls for comparison with the Asian strains from the *L. d. asiatica* (China, Korea, and Russia) and *L. d. japonica* (Japan) subspecies (subspecies designations based on [7]). None of these populations has gone through a bottleneck (no pathogens or environmental issues) since being established in our laboratory. No additional collections were made to supplement the populations or add genetic diversity. Quality control data are collected every generation to monitor for shifts in biology and the populations have maintained their distinct characteristics. Egg masses were transported to the United States Department of Agriculture, Forest Service quarantine facility in Ansonia, CT under valid permits and with permissions from the countries of origin. Voucher specimens for each population were deposited at the Entomology Division, Yale Peabody Museum of Natural History, New Haven, CT, USA.

The *L. dispar* moths that produced the egg masses used in the studies were reared in walk-in environmental chambers maintained at 25 °C, 60% Relative humidity (RH), and under a photoperiod of 16:8 (light [L]:dark [D]) hours. Larvae were reared in cohorts of 8–10, as previously described [27]. The high wheat germ artificial diet [28] was optimized for the individual populations using Wesson salt mix without iron and by adding 0.21 g (Asian populations) or 0.13 g (European and North American populations) amorphous FePO_4_ per liter of diet. Pupae were harvested, sexed, and stored by sex and population. Adults were randomly mated in groups of 25 within each population. The females oviposited on paper sheets, and eggs were harvested 40 days after mating.

Eggs were held at 5 ± 1 °C and ~100% RH with L:D of 16:8 for a minimum of 90 days to ensure that diapause requirements were met. All eggs produced by 100 individual females from each geographic population were mixed together, as is the normal process for maintaining these colonies. From the pool of mixed eggs, packets of about 500 eggs were made. Eighteen to 28 egg packets from each population were incubated over a period of five weeks to ensure that newly hatched larvae were available for use over the three weeks of setup. Eggs were incubated at 25 ± 1 °C and 60% RH with L:D of 16:8 to initiate hatching. A 0.5 g sample of eggs from each population was incubated each week to determine the embryonation and percentage hatch for each group since they received different lengths of chill prior to incubation.

Once enough larvae had hatched, melanized, and consumed any remaining yolk (as evidenced by the appearance of first frass), larvae were moved with a fine brush into numbered and pre-weighed 50 mm diameter × 9 mm deep tight-fitting petri dishes (Falcon™ 1006, Corning, Corning, NY, USA). Ten larvae were placed in each dish, the larvae and dish were weighed together, and then the weight of the dish was subtracted to determine the weight of the larvae. By dividing by ten, the average weight of the individual larvae in each dish was obtained. The dishes of larvae were then placed in clear 355 mL plastic cups (Solo TP12 cold drink cup, Solo Cup Operating Corp., Dallas, TX, USA), which were held in large clear plastic boxes on a screen over water and kept at 10 ± 1 °C and 100% RH with L:D of 16:8 until use. This prevented the larvae from desiccating and dying while waiting to be used. Additionally, gypsy moth larvae generally spend a few days resting near the egg mass before searching for food, so this ensured that they would be ready to begin feeding when placed on foliage. When possible all the larvae used for setup on a given day had been in chill for the same length of time (i.e., experienced the same duration of starvation) since this could influence the time to initiate feeding.

On the day of setup, dishes of larvae from each population were randomly assigned to each foliage type or artificial diet. Dead larvae were replaced with extra larvae from the same hatch removal date. In previous studies, the weight variation between newly hatched larvae from the same population was minimal, so only varying the time waiting in chill would likely not affect the weight of the larvae at setup.

### 2.2. Foliage Setup and Rearing

Foliage was obtained from either potted or recently planted young trees (0.6–2.0 m in height) or larger maturing trees (>3.0 m in height) for which permission to clip foliage had been obtained (Table 2). The timing of the setup for the broadleaf species was determined by foliage bud-break and the potted conifers also had new foliage when setup occurred. Short branch tips with foliage were clipped from the trees. Clippings from conifers included both the present and previous year’s foliage, and flower buds/cones if available. Clippings from broadleaf trees included both foliage and flowers if available. For each foliage change, all branch tips came from the same larger tree or randomly assigned smaller tree and had foliage/flowers of similar size and age. Over the course of the study, different trees were used and the phenological condition of the foliage/flowers changed. The ends of the clipped branches were kept in water until used, and any insects or debris found on the foliage were removed or washed off with water. If the leaves were washed, they were allowed to dry before being used. The broadleaf hosts *Quercus velutina* Lamarck and *Betula populifolia* Marshall served as preferred host controls, while *Acer rubrum* L. served as a poor host control [13]. The conifer hosts (Table 2) were chosen because they are economically or ecologically important in North America and came from several genera and from different ecosystems distributed over a wide geographic range.

Larvae were set up in clear plastic 355 mL cups (see visual abstract for photo of setup). The opening of the cup was covered with a fine mesh cloth, which was held in place by a plastic lid with a 5.5 cm hole cut out of the center. Half of a paper towel was folded and placed in the bottom of the cup so that it went up about halfway on each side. The paper towel served two purposes; providing a means for larvae that dropped off the foliage to climb back up, and an easy way to lift the water reservoir out of the cup. The paper towel was replaced each time foliage was changed. The ends of each branch tip were inserted through a 5 cm square piece of parafilm (Bemis Company, Inc., Neenah, WI, USA) covering a hole in the plastic lid of a 59 mL plastic cup (Solo TP200 soufflé cup, Solo Cup Operating Corp., Dallas, TX, USA) that was used as a water reservoir. The branch tip was re-cut while held in a 59 mL cup filled with water, then the lid was snapped onto the cup. One or two branch tips were placed in each 59 mL cup depending on the amount of foliage the larvae required. All containers of a given host plant received the same number of branch tips at each foliage change. A total of 100 larvae from each strain were set up on each host, 20 in each of five containers. All containers for each host species were set up on the same day except for Eastern white pine (*Pinus strobus* L.), which was set up across all three weeks, two containers per host per week, for a total of 120 larvae per strain. Along with the artificial diet, this provided another control to determine if larvae from the weekly hatch groups differed in quality. This species was chosen because the needle quality should not differ much over the three-week setup period and this host was one that would be used to rear larvae to adult. The plastic cups were held, 30 per tray, on rearing carts which were placed in chambers maintained at 25 ± 1 °C and 60% RH with L:D of 16:8. At each foliage change, the trays were rotated so that they spent an equal amount of time at each level on the cart.

When the larvae reached the third instar they were moved to larger containers so that more foliage could be provided. Unwaxed double-wrapped paper buckets were used (Solo 5T1-NO195 2.5-liter for hosts that went to 14 days, Solo 10T1-NO198 4.9-liter for hosts that went to pupation, Solo Cup Operating Corp., Dallas, TX, USA). Containers were covered with 0.0508 mm thick clear plastic secured with a rubber band, allowing light to enter the container. The foliage (multiple branch tips) was held in a 236 mL plastic water reservoir (Fisher 14-955-115A cup with snap-on lid, Fisher Scientific, Pittsburgh, PA, USA), and a paper towel was placed in the bottom of the container to collect frass and absorb excess water. Containers were placed, six per tray, on rearing carts which were placed in chambers maintained at 25 ± 1 °C and 60% RH with L:D of 16:8. At each foliage change, the trays were rotated so that they spent an equal amount of time at each level on the cart.

Initially, foliage was changed once per week, or more often if it was all consumed or began to dry or change color. For larger larvae, broadleaf foliage (which included the *Larix* sp.) was changed Mondays, Wednesdays, and Fridays. Coniferous foliage was changed Tuesdays and Fridays. The timing of foliage changes was based on logistical considerations and previous experience indicating that coniferous foliage is more stable than broadleaf foliage under these conditions.

For foliage changes, old foliage was replaced with freshly cut foliage in a clean water reservoir and larvae were moved to the fresh foliage. Used water reservoirs were cleaned with a weak disinfectant solution, rinsed with water, and dried before they were reused. Every seven days, the number of surviving larvae was recorded. Any dead larvae were removed, recorded, and placed in a sterile container. If larvae appeared to have been killed by a pathogen, all contaminated surfaces were removed (foliage) or replaced (container or water reservoir). Dead larvae were refrigerated until they could be assessed for the presence of pathogens. Subsequent foliage changes repeated this pattern, until either the test for that host plant ended at 14 days or all larvae had pupated or died. As the larvae grew, they were split into additional containers as needed to maintain a maximum of 15 larvae per container to prevent the larvae from consuming all the foliage before the next scheduled foliage change. Two or more branches in a water pick were added to a container if the larvae had consumed more than 60% of the foliage on a single branch between foliage changes. All containers of the same host foliage received the same number of branches at each foliage change.

The weight and instar of each larva were recorded after the larvae had been on the host foliage for 14 days. Larvae from six hosts (Table 2) were returned to rearing containers, given fresh foliage, and allowed to continue development. All larvae from the 14-day hosts were frozen.

### 2.3. Artificial Diet Rearing

A portion of the larvae from the hatch used for each week of setup on host foliage was reared on population-specific artificial diet as a control to determine if the differences in amount of chill or handling resulted in differences in larval development or survival. Ten larvae per population per week of setup were placed in each of three 177-ml clear squat plastic cups (Solo ME6RX, Solo Cup Operating Corp., Dallas, TX, USA) with unwaxed paper lids. The cups were put on trays and held at 25 ± 1 °C and 60% RH with L:D of 16:8. Weekly survival was recorded, as well as individual weight and instar at 14 days.

### 2.4. Pupae and Adults

Pupae were removed every Monday, Wednesday, Friday, and Sunday from broadleaf foliage and every Tuesday, Thursday, Friday, and Sunday from coniferous foliage. Newly formed pupae were removed from the container if possible and kept with their webbing intact in labeled 237 mL paper cups (Solo U508N, Solo Cup Operating Corp., Dallas, TX, USA); these pupae were weighed the next day. All other pupae were weighed individually the day they were found and any pupal deformities were recorded. Weighed pupae were held by population, host species, and sex in 1.6-liter containers (Solo 3T1, Solo Cup Operating Corp., Dallas, TX, USA). Pupae were checked daily, adults removed, and eclosion recorded. Females were placed individually, while males were held in groups of up to five in 237 mL paper cups labeled with population, host plant, and eclosion date. Individual pair matings within host plant species and population were set up in the females’ cups. Males were chosen randomly from those that eclosed from that host plant and population combination; males older than six days were not used. Approximately two weeks after mating, all eggs laid by an individual female were gently scraped from the cup, weighed, and placed in a glassine envelope. Around 40 days after the egg mass was laid, a sample of approximately 100 eggs (or the whole egg mass if very small) was taken and chilled at 5 ± 1 °C and ~100% RH with L:D of 16:8 for 150 days then incubated at 25 ± 1 °C and 60% RH with L:D of 16:8 to induce hatch. Percentage hatched of the embryonated eggs in each sample was determined. Fecundity and percentage embryonation (eggs turn dark brown when embryonated, while unembryonated eggs are yellow to tan) were determined by counting and evaluating all eggs in the egg mass. Only egg masses that had hatching were included in the fecundity analyses.

### 2.5. Data Analysis

The fit of each data set to various distributions was evaluated using PROC UNIVARIATE with the histogram option [29]. The Shapiro-Wilk and Anderson-Darling tests were used to assess normality. However, in cases where no distributions met the normality assumption at α = 0.05, the distribution that most closely emulated the data based on an assessment of the residuals after running the model was used. The following dependent variables were analyzed in PROC GLIMMIX [29]: time to pupation, pupal weight, time to adult, fecundity, and percentage hatch of embryonated eggs for hosts on which larvae completed development; and initial larval weight, 14-day weight gain, and percentage survival for all hosts. Population, host, sex (for pupal and adult parameters only), and all possible interactions between the three (or two) as fixed effects, and container nested within host as a random effect, were evaluated. The 14-day weight of larvae set up across different weeks on artificial diet and eastern white pine were compared to test for any larval quality differences between hatch groups. The experimental design for this part was a nested completely randomized one. The values for each container the larvae were held in were used to compare percentage survival at 14 days. The distributions and link functions used in PROC GLIMMIX [29] for each parameter were as follows: larval weight data and time to pupation were evaluated using a gamma distribution with a log link function, all data that were a percentage were evaluated using the beta distribution with a logit link function, and the other parameters were evaluated using the normal distribution with an identity link function. When the beta distribution was used, a value of 1 was replaced with 0.9999 and 0 with 0.0001, because the distribution does not allow those values. The experimental design for this part was a completely randomized one. For each model, residuals were evaluated for normality and the homogeneity of variance. Differences among means were determined by the least squares means test with α = 0.05 and a Tukey-Kramer grouping [29]. Variance differences among means were determined by the least squares means test with α = 0.05 and a Tukey-Kramer grouping [29].

## 3. Results

### 3.1. Week-of-Setup Controls

Population (*F* = 28.9; df = 5, 465; *p* < 0.0001) had a significant effect on larval weight at 14 days but neither the week of setup on artificial diet (*F* = 0.21; df = 2, 465; *p* = 0.809) nor the interaction between population and week did (*F* = 1.85; df = 10, 465; *p* = 0.0504). The week of setup on *P. strobus* (*F* = 8.97; df = 2, 275; *p* = 0.0002) and the population (*F* = 3.71; df = 5, 275; *p* = 0.0029), had a significant effect on larval weight at 14 days, but not the interaction between population and week of setup (*F* = 1.82; df = 10, 275; *p* = 0.0570). Within a population, only the first and second weeks of setup for the population from Greece differed in larval weight gain. This suggests that the differences in the time in chill the eggs received and the time the larvae were held in chill before use had minimal effects on the larvae. The mean hatch weight of larvae varied significantly by population (*F* = 682.13; df = 5, 4802; *p* < 0.0001) and host (*F* = 738.77; df = 15, 4802; *p* < 0.0001), with a significant interaction between population and host (*F* = 55.99; df = 65, 4802; *p* < 0.0001); as a consequence, hatch weight was subtracted from the weight at 14 days, and weight gain was used in the analyses. 

### 3.2. Development and Survival on Hosts at 14 Days 

**Figure 1:**
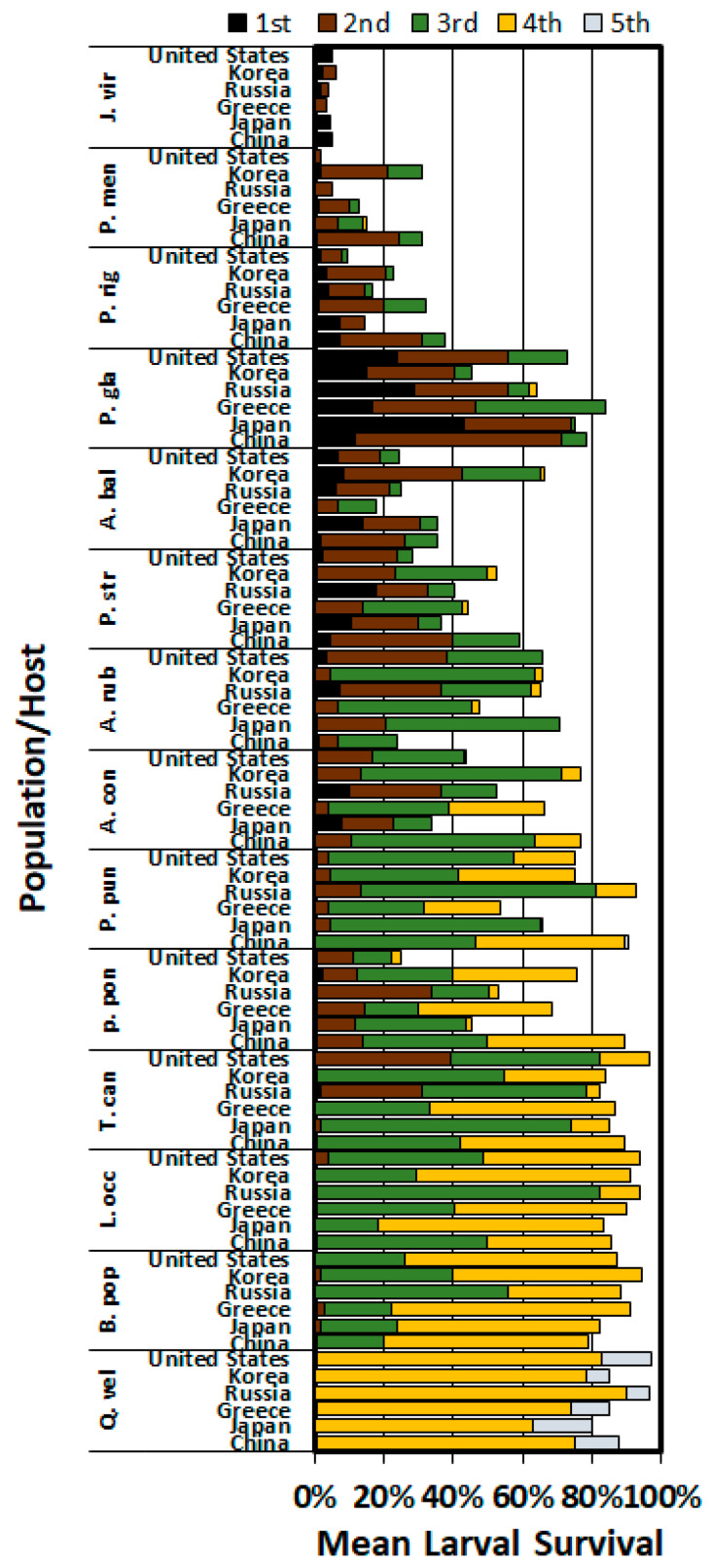
The proportion of the surviving larvae in each instar from six geographic populations on the foliage of 14 different host species. Host information is given in Table 2.

Figure 1 provides an assessment of the survival and developmental stage (instar) of larvae after 14 days on each host. On a suitable host (e.g., *Q. velutina*) the larvae should have been in the fourth or fifth instar and survival should be >50%. On some of the intermediate hosts survival was >50%, but development was slower. There were some hosts where survival was low (<50%) but the surviving larvae were still able to develop. The only hosts where no larvae molted to the second instar within 14 days were *P. palustris* and *P. taeda*, with only one Russian and one Korean larva alive on *P. palustris* and *P. taeda*, respectively (data not shown).

Population (*F* = 14.14; df = 5, 4473; *p* < 0.0001), host (*F* = 293.86; df = 15, 4473; *p* < 0.0001), and the interaction between population and host (*F* = 2.37; df = 65, 4473; *p* < 0.0001) all had significant effects on larval weight gain at 14 days. Table 3 summarizes the average weight gains for the larvae at 14 days and the population comparisons within a host. Larval weight gain did not vary significantly among populations for larvae reared on *J. virginiana, P. menziesii*, *P. pungens*, *P. rigida*, or *P. strobus*. Populations of Asian origins did not consistently gain more weight than those of European or North American origins when reared on the hosts where significant differences were observed. 

Population (*F* = 6.07; df = 5, 390; *p* < 0.0001), host (*F* = 88.92; df = 15, 390; *p* < 0.0001), and the interaction between population and host (*F* = 3.13; df = 75, 390; *p* < 0.0001) all had significant effects on larval survival at 14 days. Table 4 summarizes the average survival of larvae at 14 days and population comparisons within each host. Larval survival did not vary significantly within host by population for larvae reared on *J. virginiana, L. occidentalis*, *P. palustris*, *P. rigida*, *P. strobus*, *P. taeda*, or *T. canadensis*. On the hosts where significant differences were observed, more larvae from populations of European or North American origins consistently survived than those of Asian origins.

### 3.3. Development and Survival to Adult on Six Hosts

**Figure 2:**
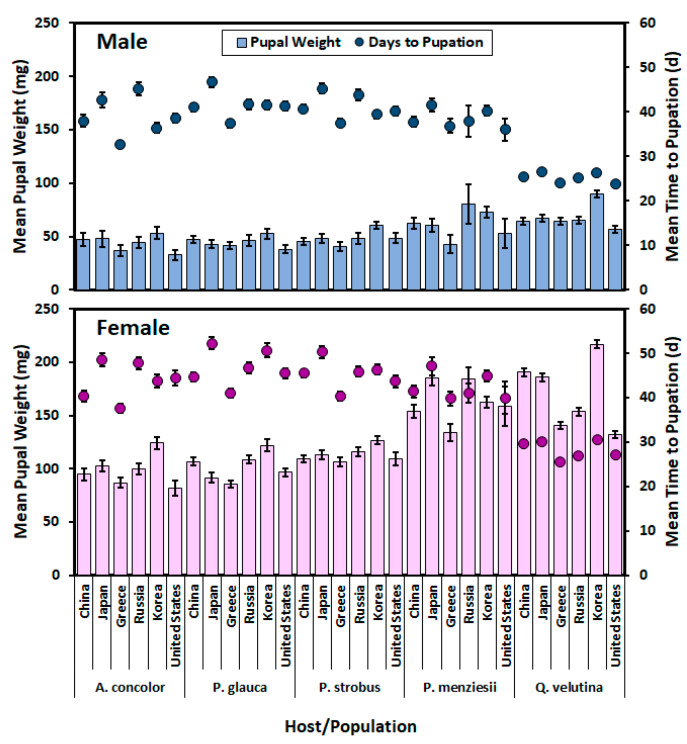
Mean (± **standard error** [SE]) pupal weights (g and bars) and time to pupation (d and dots) by sex for individuals from different source populations on each host. Top graph males and bottom graph females.

The interaction between population, sex, and host (*F* = 3.59; df = 20, 1248; *p* < 0.0001) had a significant effect on pupal weight. Females within a population were significantly larger than males from the same population on all hosts (Figure 2). North American males reared on *A. concolor* were significantly smaller than Russian and Korean males reared on *P. menziesii* and Japanese and Korean males reared on *Q. velutina*. Korean females reared on *Q. velutina* were significantly larger than Greek, Russian, and North American females reared on *Q. velutina* and females reared on all other hosts (except Japanese, Russian, Korean, and North American females on *P. menziesii).* Chinese and Japanese females reared on *Q. velutina* were larger than North American females reared on *Q. velutina* and females reared on all other hosts except *P. menziesii.*

Significant interaction between population and sex (*F* = 4.70; df = 5, 1258; *p* = 0.0003) and between host and population (*F* = 2.74; df = 20, 1258; *p* < 0.0001) were observed with respect to effects on days to pupation (Figure 2). Time to pupation was significantly shorter on *Q. velutina* than on all other hosts, and Greek individuals grew faster on *Q. velutina* than did Japanese or Korean individuals. The only other significant difference in time to pupation within a host was that Greek individuals grew faster than Japanese, Russian, and North American individuals on *A. concolor*. Females reared on *Q. velutina* grew faster than females on all other hosts, except Greek females on *A. concolor* and *P. menziesii* and North American females on *P. menziesii*. Greek females on *Q. velutina* grew faster than Chinese, Japanese, and Korean females on that same host. Chinese, Greek, Russian, and North American males grew faster on *Q. velutina* than males on all other hosts. The only within-host difference in male development was that Greek males on *A. concolor* grew faster than Japanese and Russian males on that host. The only population with no difference in the time to pupation between the sexes was the Russian population.

The interactions between population and sex (*F* = 3.96; df = 5, 1343; *p* = 0.0014), host and population (*F* = 7.66; df = 20, 1343; *p* < 0.0001), and host and sex (*F* = 2.62; df = 4, 1343; *p* = 0.0333) all had significant effects on days to adult (Table 5). Within a host there were no significant differences in time to adult eclosion for females from different populations, and the only differences in males within a host were that Greek males developed faster than Russian and Japanese males on *A. concolor* and *P. glauca*, respectively. Males reared on *Q. velutina* tended to develop faster, but the significant differences were fewer than were seen for females.

**Figure 3:**
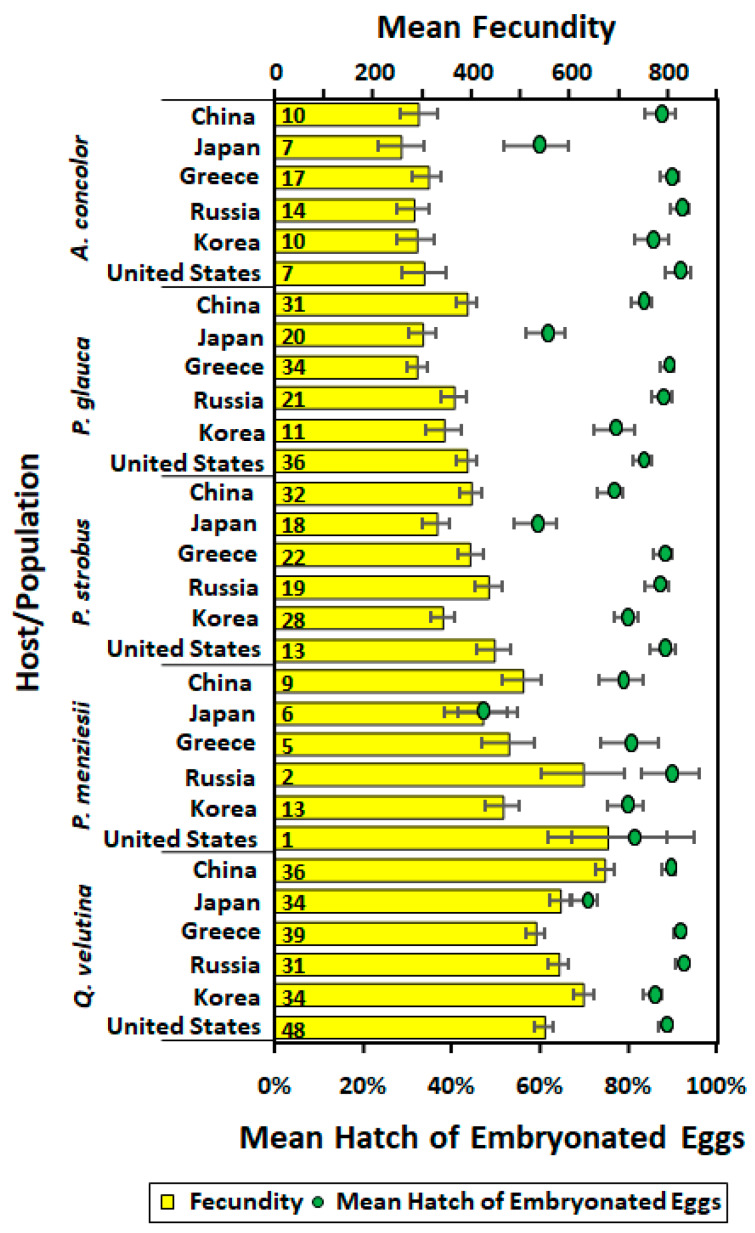
Mean (± SE) fecundity (bars) and percentage hatch of embryonated eggs (dots) for individuals from different source populations on each host. Number of females for each host and population combination are given in the bars.

Population (*F* = 4.20; df = 5, 579; *p* = 0.0009), host (*F* = 129.56; df = 4, 579; *p* < 0.0001), and interaction between population and host (*F* = 2.39; df = 20, 579; *p* < 0.0001, but no means were significantly different) all had significant effects on fecundity (Figure 3). The highest fecundity was seen in females that were reared on *Q. velutina* and *P. menziesii*, with fecundity significantly lower for the other hosts in this order: *P. strobus* > *P. glauca* > *A. concolor*. Population (*F* = 28.02; df = 5, 578; *p* < 0.0001) and host (*F* = 8.62; df = 4, 578; *p* < 0.0001) both had significant effects on percentage hatched of embryonated eggs, but the interaction between population and host did not (*F* = 0.69; df = 20, 578; *p* = 0.8427) (Figure 3). Percentage hatched of embryonated eggs laid by females from the Japanese population, regardless of host, was significantly lower than that of Russian and North American females reared on *A. concolor* and Greek and Russian females reared on *Q. velutina*. Mating success (egg masses with embryonated eggs) only dropped below 80% for Japanese females on *A. concolor* and Russian females on *P. strobus*.

## 4. Discussion

While there was variation in the ability of gypsy moth larvae from different geographic origins to utilize (survive and develop on) key North American conifers, that variation was not consistent within gypsy moth subspecies, but instead was more consistent with populations from different origins being preadapted to utilize different hosts and having different biological traits, such as speed of development and average size. For example, some Asian populations developed and survived well on some hosts, while others did not. In addition, *L. dispar dispar* populations (Greece and the Untied States.) gained weight faster and/or survived better than some *L. dispar asiatica* or *L. dispar japonica* populations on some conifers, but not all. Time to adult differed between some hosts but there was almost no significant variation between populations within a host. Female pupae from the Korean, Japanese, and Chinese populations tended to be larger, which is consistent with earlier work on these same populations [30]. Significant variation in fecundity was tied more to larval host than population, with the highest fecundity for females reared on *Q. velutina* and *P. menziesii*. Percentage hatched of embryonated eggs was not strongly affected by host, but the Japanese population had consistently lower rates of hatching than the other populations.

The results for both the hosts classified as highly favored/susceptible (*Q. velutina* and *B. populifolia*) and avoided/immune (*P. taeda* and *J. virginiana*) were consistent with previous findings for *L. dispar dispar* and varied little between populations [4,13,31,32]. On favored hosts, larvae grew faster and pupae were larger than on less preferred hosts, and on avoided hosts first instar mortality was very high. Larval development, although variable between populations, was slower and mortality higher on the intermediate/resistant hosts (i.e., *A. rubrum*, *P. strobus*, *T. canadensis*) than on the preferred hosts, as previously documented for gypsy moths from different subspecies [25,32,33]. There was disagreement between our results and some findings in the literature on three hosts: *A. balsamea*, *P. pungens*, and *P. rigida*. All previous findings for *A. balsamea* indicated that this host was avoided or resistant to gypsy moth, but in this study the larvae developed and survived well on new foliage [4,13]. In one previous study, all first instars on *P. rigida* died while in another study some larval development occurred as it did in this study [13,25,31]. *Picea pungens* was found to be one of the most suitable hosts (>80% survival of firsts) in one study, as it was here, but resistant in another [13,15]. These differences observed may be due to differences in the populations evaluated, but are more likely due to host phenology differences (availability of new foliage/buds or flowers/cones for first instar larvae to feed on), as host phenology and foliage physical characteristics can be as important to host utilization as the secondary compounds found in the leaves.

Although development was slower and survival poorer on several of the conifers, first instar larvae were able to utilize conifers unless only tougher needles were available (*P. taeda* and *P. palustris*) or feeding deterrent compounds were present (*J. virginiana*). Host phenology was also critical on some conifers since the early instars preferentially fed on new foliage or buds of *A. balsamea*, *A. concolor*, *P. strobus*, *P. glauca*, *P. ponderosa*, *P. pungens*, *P. rigida*, and *P. menziesii*. Survival of first and second instars on these hosts would have been poor if the larvae did not have soft new foliage to feed on, as has been previously found (e.g., *P. menziesii* [34]). Survival would also likely have been lower on the broadleaf control hosts, *B. populifolia* and *Q. velutina*, if the catkins they preferentially fed on were not present when the leaves were just expanding. Even though gypsy moths can feed on hundreds of hosts, the larvae may not hatch in synchrony with the most suitable hosts, so they must survive long enough to disperse and find host foliage or flowers/cones they can utilize. The length of time larvae can survive without food decreases from around a month at 5 °C to only a few days at 30 °C, but they can balloon or crawl as small larvae to find food [26,35]. In addition, there is evidence that larvae from populations without flight-capable females respond to host odors and balloon more than those from populations with flight-capable females, so this may aid larvae in the process of finding suitable food sources [36]. Fight-capable females likely lay their eggs to maximize both egg survival and ability of larvae to reach suitable host.

Although the host range of gypsy moths is known to increase at the fourth instar [4,13,31], there were two hosts that had adverse effects on larvae starting around the third instar. On *L. occidentalis* the larvae started to avoid the foliage and chew the paper container or parafilm at around 12 days when they were third or fourth instars. This agrees with previous findings that *Larix laricina* (Du Roi) K. Koch contains larval antifeedant compounds (terpenoids) and that *L. dispar dispar* larvae stopped feeding on *Larix decidua* Miller at the fourth instar [31,37]. Larvae on *A. concolor* also had issues that were more pronounced at later instars; larvae had runny frass, many died, and dead larvae liquified quickly, beginning at about the third instar. Initially it was thought that the larvae must be dying from an infection but after careful dissection and analysis, no microbial agent was found. The adverse effect of *A. concolor* on larvae was likely the result of some allelochemical that is unique to or in higher concentrations in this host. Gypsy moth is a tannin-adapted species, but the larvae are sensitive to some non-tannin phenolics (e.g., glycosides) as well as alkaloids that are found in Pinaceae [38,39,40,41,42,43]. There was also some apparent variation in the adverse effects of individual trees used, a phenomenon that has been documented for other gypsy moth hosts [44].

The local adaptation of populations to their native or introduced habitats and laboratory adaptation also may have played a role in the population differences in host utilization observed in this study. The Russian population is from a region where gypsy moths outbreak in a broadleaf forest containing few conifers, while the Greek population originated in an area where evergreen *Quercus* and *Pinus* trees predominate, with *Abies* and other conifers present at higher elevations nearby [19,45]. This may in part explain why the Russian population did not develop and survive as well as the Greek population on many of the conifers in this study. There also appear to be differences between North American gypsy moth populations both temporally and geographically, since the North American population larvae developed and survived better on some conifers than was documented in earlier studies using populations from New England, and about the same as the population collected in Oregon [13,15,31]. The results obtained in this study should, however, be interpreted with caution since several of the populations have been in laboratory culture for a number of generations, and adaptation may have occurred despite all the precautions taken to avoid it. Also, using smaller trees that may have somewhat different phenology (i.e., new foliage present earlier than in nature) and secondary compounds (differences in quality and quantity) compared to more mature trees may have impacted the ability of the larvae to use some of the hosts.

These results suggest that the measures being taken to prevent establishment of gypsy moths of all subspecies in western North America is warranted given the risk to the economically important conifers found there. This is especially true since conifers do not tolerate defoliation as well as broadleaf trees and the larvae are wasteful feeders on many conifers, consuming only part of the needle and letting the rest drop. The risk of gypsy moth establishment on southern pines appears to be lower, but larger larvae may still damage them, since needle toughness would not be a barrier. Fourth instar larvae of each population put on *P. taeda* foliage developed and survived well (unpublished data). The fact that gypsy moths from many of the Eurasian populations were able to utilize North American hosts they had never been exposed to underscores the plasticity of the phenotypes present and their ability to deal with potentially novel host chemistry, as was previously documented [3]. 

## 5. Conclusions

Populations of gypsy moth from all subspecies are polyphagous herbivores that can utilize a broad range of hosts, including many conifers. Gypsy moth larvae can utilize many novel hosts, while generally avoiding hosts with certain allelochemicals or physical traits, at least as first instars. Thus, previous assertions that the Asian subspecies pose a greater risk in terms of the hosts they can utilize appears to be incorrect. The gypsy moth is a very adaptable invasive species that warrants taking measures to prevent its spread, regardless of subspecies. 

## Figures and Tables

**Table 1:** Subspecies and approximate location (latitude and longitude) of source populations, evaluated in this study.

Subspecies	Country	Closest City, Region	Collection Date ^a^	Egg Masses Received	Latitude	Longitude
*Lymantria dispar asiatica*	China	Harbin, Heilongjiang	Aug-2012 (Oct-2012)	6 individual	45.78° N	126.61° E
*Lymantria dispar asiatica*	Russia	Mineralni, Primorski	Aug-1992	20 individual	44.10° N	133.15° E
*Lymantria dispar asiatica*	Korea	Samhwa-Dong, Gangovon-Do	Aug-2009 (Nov-2014)	16 individual	37.49° N	129.06° E
*Lymantria dispar japonica*	Japan	Takizawa, Morika, Nishine Hachimantai City, Northern Iwate District, Honshu	Oct-2005 (Nov-2014)	15 individual	39.73° N	141.08° E
*Lymantria dispar dispar*	Greece	Kavála, Macedonia	Feb-1997	58 individual	41.00° N	24.25° E
*Lymantria dispar dispar*	United States	Bethany, New Haven County, CT	Mar-1994	12 individual	41.25° N	73.00° W

^a^ Date in parentheses is when the population was received at the Forest Service Quarantine Laboratory in Ansonia, CT. All populations start each laboratory generation as a mixture of 100 egg masses from the previous generation and the majority of individuals that pupate each generation are mated to maintain genetic diversity.

**Table 2:** Foliage sources for each tree species used and information about the setup of the study.

Duration on Host	Julian Day Setup	Host Family	Host Genus	Host Species	Authority	Host Common Name	Host Region	Foliage Source Location ^a^	Host Height (n)
14 Days	124	Aceraceae	*Acer*	*rubrum*	Linnaeus	Red Maple	E	Hamden, CT	25 m trees (5)
14 Days	116	Betulaceae	*Betula*	*populifolia*	Marshall	Gray Birch	E	Ansonia, CT	6 m trees (10)
14 Days	123	Cupressaceae	*Juniperus*	*virginiana*	Linnaeus	Eastern Red Cedar	E	Ansonia, CT	10 m trees (5)
Pupation	130	Fagaceae	*Quercus*	*velutina*	Lamarck	Black Oak	E	Ansonia, CT	25 m trees (5)
14 Days	118	Pinaceae	*Abies*	*balsamea*	(L.) Miller	Balsam Fir (Canada)	N	Vans Pines Nursery	0.3 m plugs (32)
Pupation	117	Pinaceae	*Abies*	*concolor*	(Gordon) Lindley	White Fir (Santa Fe)	W	Vans Pines Nursery	0.6 m potted (51)
14 Days	120	Pinaceae	*Larix*	*occidentalis*	Nuttall	Western Larch	W	Ansonia, CT	2 m trees (4)
Pupation	125	Pinaceae	*Picea*	*glauca*	(Moench) Voss	White Spruce	N	Vans Pines Nursery & Derby, CT	0.6 m potted (25) & 30 m trees (10)
14 Days	131	Pinaceae	*Picea*	*pungens*	Engelmann	Colorado Blue Spruce	W	Hamden, CT	30 m tree (1)
14 Days	127	Pinaceae	*Pinus*	*palustris*	Miller	Longleaf Pine	S	Nature Hills Nursery	1.2 m potted (3)
14 Days	126	Pinaceae	*Pinus*	*ponderosa*	Douglas	Ponderosa Pine	W	Vans Pines Nursery	0.6 m potted (25)
14 Days	132	Pinaceae	*Pinus*	*rigida*	Miller	Pitch Pine	NE	Van Pines Nursery	0.6 m potted (27)
Pupation	116, 124, 133	Pinaceae	*Pinus*	*strobus*	Linnaeus	Eastern White Pine	E	Ansonia, CT	25 m trees (5)
Pupation	119	Pinaceae	*Pinus*	*taeda*	Linnaeus	Loblolly Pine	S	Pineville, LA	30 m trees (4)
Pupation	134	Pinaceae	*Pseudotsuga*	*menziesii*	(Mirbel) Franco	Douglas Fir (Blue)	W	Vans Pines Nursery & Hamden, CT	0.4 m potted (42) & 25 m trees (2)
14 Days	133	Pinaceae	*Tsuga*	*canadensis*	(L.) Carrière	Eastern Hemlock	E	Ansonia, CT	2 m trees (5)

^a^ Vans Pines Nursery, West Olive, MI plugs (*A. balsamea* only) or potted plants; Nature Hills Nursery, Omaha, NE potted plants; Pineville, LA host material was cut and shipped overnight, all other host material was cut locally (landscape or greenhouse trees) and used the same day.

**Table 3:** Mean ± SE weight gain (mg) (n) of larvae from different source populations after 14 days on each host.

Host	China	Japan	Greece	Russia	Korea	United States	Statistics
***A. balsamea***	**8.0 ± 2.0 ab (34)**	**5.0 ± 1.0 b (36)**	**8.0 ± 2.0 ab (20)**	**4.0 ± 1.0 b** **(29)**	**13.0 ± 3.0 a (68)**	**7.0 ± 2.0 ab (25)**	**F = 2.96; df = 5, 182;** ***p* = 0.0136**
***A. concolor***	**26.0 ± 4.0 ab (80)**	**10.0 ± 2.0 cd (37)**	**44.0 ± 7.0 a (68)**	**8.0 ± 1.0 d** **(53)**	**32.0 ± 5.0 ab (81)**	**18.0 ± 3.0 bc (45)**	**F = 14.68; df = 5, 333; *p* <0.0001**
***A. rubrum***	**28.0 ± 4.0 ab (48)**	**35.0 ± 4.0 a (75)**	**32.0 ± 5.0 a (47)**	**16.0 ± 2.0 b (68)**	**35.0 ± 5.0 a (68)**	**17.0 ± 2.0 b (69)**	**F = 7.17; df = 5, 346;** ***p* <0.0001**
***B. populifolia***	**121.0 ± 11.0 a (79)**	**118.0 ± 10.0 a (82)**	**126.0 ± 11.0 a (93)**	**82.0 ± 7.0 b (90)**	**128.0 ± 11.0 a (90)**	**110.0 ± 9.0 ab (89)**	**F = 3.67; df = 5, 493;** ***p* = 0.0028**
*J. virginiana*	0.4 ± 0.3 a (3)	1.3 ± 0.8 a (7)	2.1 ± 2.0 a (4)	1.3 ± 0.9 a (2)	1.7 ± 0.9 a (11)	0.4 ± 0.3 a (3)	F = 0.89; df = 5, 15; *p* = 0.5095
***L. occidentalis***	**56.0 ± 3.0 c** **(87)**	**104.0 ± 5.0 a (87)**	**63.0 ± 3.0 bc** **(91)**	**43.0 ± 2.0 d (88)**	**100.0 ± 5.0 a (93)**	**73.0 ± 4.0 b (89)**	**F = 51.00; df = 5, 505; *p* <.0001**
***P. glauca***	**8.0 ± 1.0 ab** **(80)**	**5.0 ± 1.0 b (75)**	**11.0 ± 2.0 a** **(85)**	**7.0 ± 1.0 ab (62)**	**9.0 ± 1.0 ab (45)**	**6.0 ± 1.0 ab (73)**	**F = 3.84; df = 5, 387;** ***p* = 0.0021**
*P. menziesii*	11.0 ± 2.0 a (29)	11.0 ± 3.0 a (17)	8.0 ± 2.0 a (11)	9.0 ± 3.0 a (4)	11.0 ± 3.0 a (32)	14.0 ± 8.0 a (3)	F = 0.20; df = 5, 71; *p* = 0.9627
***P. ponderosa***	**39.0 ± 9.0 ab** **(95)**	**19.0 ± 5.0 b (47)**	**57.0 ± 13.0 a** **(76)**	**23.0 ± 5.0 ab (55)**	**51.0 ± 12.0 ab (66)**	**20.0 ± 5.0 b (38)**	**F = 4.04; df = 5, 348;** ***p* = 0.0014**
*P. pungens*	72.0 ± 10.0 a (84)	62.0 ± 9.0 a (67)	64.0 ± 10.0 a (54)	51.0 ± 7.0 a (91)	68.0 ± 10.0 a (78)	59.0 ± 8.0 a (76)	F = 0.69; df = 5, 420; *p* = 0.6347
*P. rigida*	6.0 ± 2.0 a (40)	2.0 ± 1.0 a (10)	3.0 ± 1.0 a (26)	2.0 ± 1.0 a (21)	2.0 ± 1.0 a (12)	4.0 ± 2.0 a (22)	F = 1.87; df = 5, 106; *p* = 0.1062
*P. strobus*	4.0 ± 1.0 a (73)	4.0 ± 1.0 a (47)	5.0 ± 2.0 a (53)	2.0 ± 1.0 a (50)	6.0 ± 2.0 a (65)	3.0 ± 1.0 a (33)	F = 2.10; df = 5, 275; *p* = 0.0655
***Q. velutina***	**337.0 ± 13.0 bc (90)**	**293.0 ± 11 c (81)**	**419.0 ± 16.0 a (86)**	**378.0 ± 14.0 ab (90)**	**347.0 ± 13.0 b (87)**	**385.0 ± 14.0 ab (95)**	**F = 10.55; df = 5, 499; *p* <.0001**
***T. canadensis***	**45.0 ± 3 a** **(91)**	**39.0 ± 3 a** **(86)**	**47.0 ± 3.0 a (88)**	**17.0 ± 1.0 b (82)**	**42.0 ± 3.0 a (86)**	**40.0 ± 3.0 a (90)**	**F = 28.78; df = 5, 493; *p* <.0001**

a–d: Weight gain was calculated as 14-day weight minus hatch weight. Means in the same row followed by the same letter are not significantly different when analyzed by PROC GLIMMIX followed by Tukey-Kramer Least Squares Mean test with α = 0.05 [29]. Rows where significant differences between populations occur are in bold text.

**Table 4:** Mean percentage survival (± SE) of larvae from different source populations after 14 days on each host.

	Gypsy Moth Population						
Host	China	Japan	Greece	Russia	Korea	United States	Statistics
***A. balsamea***	**35.4 ± 7.4 ab**	**35.7 ± 7.4 ab**	**17.8 ± 5.5 b**	**25.1 ± 6.5 b**	**66.2 ± 7.3 a**	**24.7 ± 6.5 b**	***F* = 4.92; df = 5, 24;** ***p* = 0.0031**
***A. concolor***	**76.9 ± 6.1 a**	**33.8 ± 7.1 b**	**66.3 ± 7.1 ab**	**52.8 ± 7.6 ab**	**77.0 ± 6.1 a**	**44.0 ± 7.5 b**	***F* = 5.45; df = 5, 24;** ***p* = 0.0017**
***A. rubrum***	**24.2 ± 7.5 b**	**70.7 ± 8.2 a**	**47.5 ± 9.4 ab**	**65.2 ± 8.8 a**	**65.7 ± 8.7 a**	**66.0 ± 8.7 a**	**F = 3.47; df = 5, 24;** ***p* = 0.0168**
***B. populifolia***	**79.2 ± 4.2 b**	**82.1 ± 3.9 ab**	**91.0 ± 2.7 ab**	**88.2 ± 3.2 ab**	**94.4 ± 2 a**	**87.0 ± 3.3 ab**	**F = 2.9; df = 5, 24;** ***p* = 0.0347**
*J. virginiana*	5.3 ± 2.7 a	4.6 ± 2.4 a	3.5 ± 1.9 a	4.1 ± 2.1 a	6.4 ± 3.1 a	5.3 ± 2.7 a	F = 0.26; df = 5, 24; *p* = 0.9317
*L. occidentalis*	85.3 ± 4.0 a	83.2 ± 4.2 a	89.8 ± 3.2 a	94.0 ± 2.3 a	91.2 ± 3.0 a	94.1 ± 2.3 a	F = 1.92; df = 5, 24; *p* = 0.1281
***P. glauca***	**78.2 ± 4.8 a**	**74.9 ± 5.1 a**	**84.0 ± 4.2 a**	**64.2 ± 5.8 ab**	**45.3 ± 6.0 b**	**73.0 ± 5.3 a**	**F = 5.83; df = 5, 24;** ***p* = 0.0012**
***P. menziesii***	**30.9 ± 6.5 a**	**15.0 ± 4.7 ab**	**12.8 ± 4.3 ab**	**5.4 ± 2.3 bc**	**30.9 ± 6.5 a**	**2.2 ± 1.1 c**	**F = 7.77; df = 5, 24;** ***p* = 0.0002**
P. palustris*	0.2 ± 0.1 a	0.2 ± 0.1 a	0.2 ± 0.1 a	0.2 ± 0.1 a	0.2 ± 0.1 a	0.2 ± 0.1 a	F = 0.11; df = 5, 24; *p* = 0.9901
***P. ponderosa***	**89.6 ± 4.8 a**	**45.7 ± 12.0 b**	**68.7 ± 10.6 ab**	**53.2 ± 12.1 ab**	**75.7 ± 9.2 ab**	**24.8 ± 9.3 b**	**F = 4.43; df = 5, 24;** ***p* = 0.0053**
***P. pungens***	**90.5 ± 3.7 ab**	**66.0 ± 7.2 bc**	**53.5 ± 7.6 c**	**92.6 ± 3.1 a**	**75.2 ± 6.4 abc**	**75.1 ± 6.4 abc**	**F = 5.62; df = 5, 24;** ***p* = 0.0014**
*P. rigida*	37.7 ± 11.5 a	14.8 ± 6.5 a	32.3 ± 10.8 a	17 ± 7.2 a	22.6 ± 8.8 a	9.8 ± 4.6 a	F = 1.68; df = 5, 24; *p* = 0.1778
*P. strobus*	59.4 ± 7.5 a	36.4 ± 7.3 a	44.4 ± 7.6 a	40.6 ± 7.5 a	52.5 ± 7.7 a	28.2 ± 6.7 a	F = 2.15; df = 5, 30; *p* = 0.0866
P. taeda *	0.2 ± 0.1 a	0.2 ± 0.1 a	0.2 ± 0.1 a	0.2 ± 0.1 a	0.2 ± 0.1 a	0.2 ± 0.1 a	F = 0.11; df = 5, 24; *p* = 0.9901
***Q. velutina***	**87.5 ± 3.7 abc**	**79.8 ± 4.7 c**	**84.9 ± 4.1 bc**	**96.4 ± 1.6 ab**	**85.3 ± 4.0 bc**	**97.0 ± 1.4 a**	**F = 4.79; df = 5, 24;** ***p* = 0.0036**
*T. canadensis*	89.2 ± 3.4 ab	85.0 ± 4.1 ab	86.6 ± 3.9 ab	82.2 ± 4.5 b	83.9 ± 4.3 ab	96.3 ± 1.6 a	F = 2.51; df = 5, 24; *p* = 0.0579

* Only one Russian and one Korean larva were alive on *P. palustris* and *P. taeda*, respectively, at 14 days. Because the beta distribution was used, values of 1 and 0 were replaced with 0.9999 and 0.0001, respectively. a–c: Means in the same row followed by the same letter are not significantly different when analyzed by PROC GIMMIX followed by Tukey-Kramer Least Squares Mean test with α = 0.05 [29]. Rows where significant differences between populations occur are in bold text.

**Table 5:** Mating success (percentage of matings that resulted in viable eggs) and mean (± SE) time to adult (d) separated by sex, for different source populations reared on each host.

Host	Population	Time to Adult (d)				% Mating Success
		Male	n	Female	n	
P. taeda *	Korea	62	1	NA	0	NA
*A. concolor*	China	**50.8 ± 1.2 abcd**	10	51.2 ± 1 abcd	13	83.3
*A. concolor*	Japan	**55.6 ± 1.4 abcd**	7	59.3 ± 1.1 abcd	12	77.8
*A. concolor*	Greece	**44.5 ± 1.1 cde**	11	48.3 ± 0.9 bcde	19	89.5
*A. concolor*	Russia	**56 ± 1 ab**	13	56.6 ± 1 ab	14	100.0
*A. concolor*	Korea	**49.2 ± 1 bcde**	15	54.5 ± 1 ab	13	100.0
*A. concolor*	United States	**49.6 ± 0.8 bcde**	20	52.1 ± 1.3 abc	8	100.0
*P. glauca*	China	**53.4 ± 0.6 ab**	38	55.9 ± 0.6 ab	34	100.0
*P. glauca*	Japan	**58.6 ± 0.7 a**	30	63 ± 0.8 a	22	90.6
*P. glauca*	Greece	**48.3 ± 0.6 bcde**	44	50.8 ± 0.6 abcd	35	97.1
*P. glauca*	Russia	**52.5 ± 0.9 abc**	17	56.9 ± 0.7 ab	30	95.5
*P. glauca*	Korea	**53.9 ± 0.8 ab**	21	61.3 ± 1.1 a	12	91.7
*P. glauca*	United States	**52.3 ± 0.7 abc**	30	55 ± 0.6 ab	36	100.0
*P. strobus*	China	54 ± 0.6 ab	38	56.6 ± 0.6 ab	34	97.0
*P. strobus*	Japan	58 ± 0.8 ab	23	59.9 ± 0.9 a	19	100.0
*P. strobus*	Greece	47.8 ± 0.8 bcde	24	50.5 ± 0.8 abcd	24	100.0
*P. strobus*	Russia	55.1 ± 1 ab	15	55.8 ± 0.8 ab	24	79.2
*P. strobus*	Korea	52.2 ± 0.6 abc	33	58.1 ± 0.7 ab	30	96.6
*P. strobus*	United States	51.4 ± 0.9 abcd	19	52.3 ± 1.1 abc	12	100.0
*P. menziesii*	China	51.2 ± 0.9 abcd	17	53.4 ± 1.2 ab	10	100.0
*P. menziesii*	Japan	51.9 ± 1.1 abcd	11	56.2 ± 1.5 ab	6	100.0
*P. menziesii*	Greece	47.8 ± 1.7 bcde	5	50 ± 1.7 bcde	5	100.0
*P. menziesii*	Russia	52 ± 3.7 abcd	1	53 ± 2.1 abc	3	100.0
*P. menziesii*	Korea	52.7 ± 1 abc	14	56.1 ± 0.9 ab	16	100.0
*P. menziesii*	United States	47.5 ± 2.6 bcde	2	50 ± 3.7 abcde	1	100.0
*Q. velutina*	China	37.3 ± 0.5 de	49	41 ± 0.6 de	39	92.3
*Q. velutina*	Japan	38.8 ± 0.6 de	42	41.3 ± 0.6 de	37	94.4
*Q. velutina*	Greece	34.8 ± 0.6 e	39	35.7 ± 0.6 e	41	100.0
*Q. velutina*	Russia	35.9 ± 0.5 e	50	37.6 ± 0.6 de	38	86.5
*Q. velutina*	Korea	39.3 ± 0.5 de	46	42.4 ± 0.6 de	38	91.9
*Q. velutina*	United States	34.9 ± 0.6 e	41	36.8 ± 0.5 de	53	94.1

* Only one Korean male pupated on *P. taeda*, so it was not included in the statistical analysis. a–e: Means in the same column followed by the same letter are not significantly different when analyzed by PROC GLIMMIX [29] followed by Tukey-Kramer Least Squares Mean test with α = 0.05. Time to adult values for hosts where significant between population variation occurs are in bold.
